# Metagenomics study of soil microorganisms involved in the carbon cycle in a saline–alkaline meadow steppe in the Songnen Plain in Northeast China

**DOI:** 10.3389/fmicb.2024.1335488

**Published:** 2024-03-04

**Authors:** Huichuan Xiao, Yinzhu Wei, Xuetong Sun, Xue Song, Jielin Liu, Zhenjian Bai, Guofu Hu, Ligang Qin

**Affiliations:** ^1^College of Animal Science and Technology, Northeast Agricultural University, Harbin, Heilongjiang, China; ^2^Grassland Institute of Heilongjiang Academy of Agricultural Sciences, Harbin, Heilongjiang, China

**Keywords:** saline–alkaline meadow steppe, soil microorganisms, carbon cycle immobilization-related functional genes, structural equation model, soil carbon cycle

## Abstract

Soil microorganisms play an important role in regulating and contributing to carbon cycling processes in grassland ecosystems. Soil salinization is one of the major problems causing soil degradation, and its effects on carbon cycle immobilization-related functional genes in soil microorganisms remain unknown. Therefore, we took Songnen salinization grassland as the research object, selected grasslands with different salinization levels, and explored the diversity of soil microorganisms and functional genes related to carbon cycling in Songnen grassland with different salinization levels through metagenomic technology. The results showed that with the increase of salinity, the relative abundance of *Ascomycetes* increased, while the relative abundance of Proteus and Firmicutes decreased. In addition, the relative abundance of functional genes related to carbon cycling fixation has also decreased. As the degree of soil salinization increases, the relative abundance of glycoside hydrolases (GH)130 family significantly increases, while the relative abundance of soil carbohydrate enzymes belonging to GH3 and GH55 families significantly decreases. Using structural equation modeling (SEM), it was found that soil pH and conductivity (EC) have a significant impact on soil microbial diversity and functional genes related to carbon cycling fixation. The increase in soil pH directly reduces the Shannon diversity of soil microbial diversity and functional genes related to carbon cycling fixation. Therefore, it can be concluded that the intensification of grassland salinization reduces the diversity of bacteria and fungi, and affects the diversity of functional genes related to carbon cycling fixation by reducing the total diversity of bacteria. The increase in salinity has a negative feedback effect on grassland soil carbon cycling. This study provides a theoretical framework for grassland soil carbon sequestration and degradation restoration.

## 1 Introduction

Soil salinization has become a global ecological problem, with approximately 3% of the soil resources being salinized worldwide. The total area of saline soils in China is 35 million ha; this includes 29 million ha of grassland saline soils (Mu et al., 2014). Human activities, such as overgrazing and perennial cultivation, remove vegetation, destroy the topsoil structure, increase surface evaporation and further transfer soluble salts from deeper soils, thereby increasing the salinization of grassland soils and severely inhibiting grassland productivity (Guangqiang et al., 2009; Lu et al., 2022). As an important part of the ecosystem, soil microorganisms are not only involved in the material cycle and energy transformation process of the ecosystem, but also able to respond rapidly to changes in environmental factors (Carolan and Fornara, 2016; Ma et al., 2017). Soil microorganisms can regulate the storage and release of organic carbon in soil by changing the rate of decomposing organic matter. Therefore, the population and metabolic characteristics of microorganisms are important indicators of the response of grassland ecosystems to soil salinization (Zeng et al., 2018).

Soil microorganisms play an important role in regulating and promoting the carbon cycle process in grassland ecosystems (Li et al., 2013; Tang et al., 2019). Organic matter in soil can provide carbon source to microorganisms, which is absorbed and utilized by microorganisms in decomposition. The CO_2_ produced during microbial decomposition and respiration is returned to the atmosphere. Previous studies have shown that increasing soil carbon sequestration is a very important way to actively address and mitigate global climate change (Dai et al., 2018). Among them, microbial residues are an important source of soil organic carbon. It was found that the average contribution of microbial residual carbon to organic carbon in grassland soils (0–20 cm topsoil) was 47% (Wang B. et al., 2021; Wang L. P. et al., 2021). Soil carbon export through carbon emissions (soil respiration and methane emission) caused by soil microorganisms and the leaching of dissolved organic carbon are also important determinants of the stability of the soil carbon pool (Li L. F. et al., 2021; Li C. Y. et al., 2021; Li B. et al., 2021). Various specific carbon processes are responsible for the entire carbon cycle; these processes are regulated by genes involved in catabolism (through decomposition) and anabolism (through biosynthesis) (Balasubramanian et al., 2020). Specific carbon cycle immobilization-related genes (e.g., those associated with the conversion of glucosides, starch, esters, chitin and lignin) and the microorganisms containing them are highly correlated with environmental factors, such as salinity, in grassland soils and play a more important role in soil carbon conversion and the stabilization of carbon pools than unrelated genes (Wang et al., 2013). Furthermore, the mechanisms of potential soil microbes and metabolites in response to the salinization of grassland soils remain unknown (Fornara et al., 2020). To understand the processes involved in the carbon cycle and their association with organic compounds, it is crucial to identify genes involved in these specific processes (Nelson et al., 2017).

To date, the impact of grassland salinity on soil carbon cycling has been mainly assessed by measuring the soil microbial biomass and assessing changes in the community structure. More recently, a small number of studies have focused on the effects of grassland salinization on bacterial or fungal composition (Crowther et al., 2019; Xu et al., 2021). However, in contrast to studies on soil bacterial or fungal composition, the true impact of soil salinity on microbial functional genes remains unknown. Moreover, little is known about the interrelationships among soil salinity, soil microbial (bacterial and fungal) diversity and potential functional genes (Duran et al., 2013; Taylor et al., 2021).

In this study, we used soil metagenomics to compare soil microbial genes involved in carbon cycling in different saline grasslands and to examine their response to soil and environmental factors. We chose the saline meadow steppe in the Songnen Plain in Northeast China as the ideal platform for this study because this region has the largest concentration of sodic–saline soils in China, in addition to exhibiting typical saline grassland characteristics and a clear gradient of different saline grasslands. This study aimed to (i) determine the composition of microbial carbon cycle immobilization-related genes and enzymes associated with different levels of salinity and (ii) assess the mechanisms linking the physicochemical properties of soil with microbial species diversity and functional genes involved in carbon cycling in grasslands with different salinity gradients. We hypothesized that (i) soil salinization has a significant effect on the soil carbon cycle and soil microbial genes in grasslands and (ii) an increase in grassland soil salinization decreases the microbial species diversity and carbon cycle immobilization-related functional gene diversity in the soil.

## 2 Materials and methods

### 2.1 Site description and assessment of soil characteristics

The research area is located in SuiHua station at the Chinese National Technology System of Forage Industry (E 125° 28′ 24″, N 46° 32′ 17″), which is located southeast of the Songnen Plain grassland and has a continental monsoon climate. The annual rainfall in this area is 469.7 mm, and the average annual temperature is 2.9°C. The annual accumulated temperature is ≥10°C, active accumulated temperature is 2,760°C, annual average sunshine duration is 2,713 h, annual frost-free period is 130 days and annual freezing period is 183 days (Wang B. et al., 2021; Wang L. P. et al., 2021). The soil is mainly composed of light chernozem and salinised meadow soil. The vegetation mainly consists of mesophytic or xerophytic gramineous plants. The predominant plant community is *Leymus chinensis* (Trin.) Tzvel., with *Lathyrus quinquenervius* (Miq.) Litv., *Chloris virgata* Sw., *Puccinellia distans* (L.) Parl., *Hemarthria altissima* (Poir.) Stapf et C.E. Hubb., *Carex duriusculasubsp stenophylloides* (V.I. Kreczetowicz) S. Yun Liang & Y.C. Tang, *Phragmites communis*, *Suaeda heteroptera* and other plants being present as companion species (Chen et al., 2021).

Five grassland sites with different salinity levels were selected according to the composition of different plant communities: mildly saline grassland (MIS) with *L. chinensis* (Trin.) Tzvel. as the predominant species, moderately saline grassland (MDS) with broadleaved herbs as the predominant species, heavily saline grassland (HES) with *P. tenuiflora* (Griseb.) Scribn. et Merr. as the predominant species, extremely saline grassland (ETS) with *S. glauca* (Bunge) Bunge as the predominant species and alkali spot (AS) with no vegetation. Three 10 m × 10 m plots were set up in duplicates at each site at an interval of 1 m. Composite samples were obtained by combining five topsoil samples (0–20 cm) from each plot. There was a total of 5 salinity gradients, each with 3 replicates, and one sample was collected in each cell, for a total of 15 samples. Each soil sample was divided into two groups: one was weighed directly, air dried and passed through a 2 mm sieve for assessing its physicochemical property and the other was immediately packed into a 50 mL centrifuge tube, placed in an incubator with dry ice, brought back to the laboratory, stored in a − 80°C refrigerator and then sent to Biomarker Technologies for microbial macrogenome sequencing.

SOM content was determined using the dichromate oxidation method by adding sulfuric acid (H_2_SO_4_) and titrating with ferrous ammonium sulphate. Total nitrogen (TN) content was measured using the Kjeldahl method. Total phosphorus (TP) content was measured by melted molybdenum, antimony and scandium colorimetry. Soil water content (SWC) was gravimetrically measured by drying the samples at 105°C overnight and expressed as a percentage of the dry weight. Soil pH is measured using a pH meter (PHSJ-4F, Shanghai Yidian Scientific Instrument Co., Ltd., China) in a 1:2.5 soil: water suspension. EC was determined using a conductivity meter (DDS-307, Shanghai Yidian Scientific Instrument Co., Ltd., China) after shaking an air-dried soil/water suspension (1:5, w/v) for 30 min.

### 2.2 DNA extraction and sequencing and data processing

The MoBio Power Soil DNA Isolation Kit (MoBio, Carlsbad, CA, United States) was used to extract soil DNA, according to the manufacturer’s instructions. After monitoring the quality and concentration of DNA samples using Qubit, the prepared DNA samples were sent to Biomarker Technologies Co, LTD (Beijing, China) for shotgun macrogenome sequencing using a Qubit ® 2.0 fluorometer. Paired-end reads were generated using NovaSeq 6,000 linked with the Illumina 2 × 150 bp platform. All sequences have been stored in the European Nucleotide Archive under the research registration number erp 121,142 (Zhou et al., 2021).

Quality control was performed on the original reads obtained by sequencing, and the reads were filtered to obtain clean reads for subsequent bioinformatic analysis. Clean reads were spliced and assembled, coding genes were predicted and non-redundant gene sets were constructed and subjected to functional annotation and taxonomic analysis using general and specialized databases. Statistical sample species composition and abundance data were set using default parameters, and low-quality reads were removed using Trimmomatic.^1^ Duplicate reads were removed using FastUniq (Zhou et al., 2017). MEGAHIT was then used to assemble the filtered reads via the de Bruijn graph algorithm, which presents large parameters and removes overlapping groups shorter than 500 bp. Prodigal was used to predict protein-coding genes with default settings (Ma et al., 2021). In the blastp mode, predicted genes were explored using the NCBI non-redundant protein sequence database (e value: 1e−5 and sensitivity). The abundance of EC genes associated with carbon cycle immobilization-related enzymes was quantified using MEGAN6 (version 6.18) and the Kyoto Encyclopedia of Genes and Genomes (KEGG) (version 84.1) (100% identification) (Ma et al., 2021). In addition, the gene read number for each sample was normalized based on the minimum read number. Annotated readings were associated with gene abundance (percentage).

dB CAN-seq (Database of CAZyme sequence and annotation) integrated with the HMMER tool was used for the automatic annotation of carbohydrate-active enzymes (CAZymes) (Li L. F. et al., 2021; Li C. Y. et al., 2021; Li B. et al., 2021). The degradation, modification and production of glycosidic bonds are directly related to the decomposition and biosynthesis of soil organic carbon (Hayes and Swift, 2020). The annotated genes were divided into six groups: glycoside hydrolases (GHs), glycosyl transferases (GTs), polysaccharide lyases (PLs), carbohydrate esterases (CEs), auxiliary activities (AAs) and carbohydrate-binding modules (CBMs). GH genes (EC 3.2.1.-) are involved in the hydrolysis of glycosidic bonds of glycosides. Similar to PLs and CEs, GHs decompose organic carbon, whereas AAs decompose lignin (Zhang et al., 2013). However, GT genes (EC 2.4.-) catalyze the formation of glycosidic bonds and are involved in the biosynthesis of organic carbon. The number of reads of the six groups of genes in each sample was normalized according to the minimum number of reads. The relative abundance (percentage) of specific genes was calculated based on the annotated reading. In accordance with the lowest-common ancestor algorithm, Diamond and MEGAN6 were used for the phylogenetic allocation of phylum-dominant predominant groups of genes associated with the carbon cycle from the NCBI non-redundant protein sequence database. A phylogenetic tree was constructed using MEGAN6. The phylogenetic tree included the type and abundance of taxa containing specific genes related to the carbon cycle and was visualized using the Interactive Tree of Life (iTOL) online interface (Ding et al., 2019). Gene abundance data were used to assess the microbial potential for decomposing SOM components with stubborn gradients.

### 2.3 Data analysis

R version 3.6.1 was used for all statistical analyzes. To filter the functions showing similar reactions under grassland salinization, the TPM (Transcripts Per Kilobase per Million mapped reads) matrix was aggregated based on Pfam annotations. If the average TPM abundance of four biological replicates under grassland salinization was higher than that under physical and chemical environmental conditions, one Pfam domain was considered more abundant under salinization conditions (He et al., 2019). Based on whether the parameter conditions (homogeneity of normal distribution and variance) were met, the statistical significance of the findings was evaluated using parametric or non-parametric tests (Li et al., 2019).

The adonis function was used to perform permutational multivariate analysis of variation (PERAMOVA) to determine the different saline-alkaline on the observed functional abundance and classification abundance patterns. Heat maps were generated using the pheatmap function of the pheatmap R package. Based on similar distribution patterns, rows were clustered by complete linkage. All the matrices were generated using the Canberra distance, and PERMANOVA was performed using 9,999 permutations (Chen et al., 2020). The number of unique genes and functions was calculated by iterating (*n* = 10) sparse and fixed depth replacements (75% of the sample reading with the lowest total basis factor), and the number of transcripts mapped to each gene potentially involved in organic carbon source degradation was used as the probability vector. The rmultinom and, respectively, functions in the R package were used to calculate gene and functional abundance values. The same method was used to calculate the number of unique classification groups (Klumpp, 2021).

As suggested by Bentler and Chou (1987), SEM can be used on any data conforming to a normal distribution as long as the sample size is not too large (Jiang et al., 2021). Consequently, SEM with the maximum likelihood estimation method in AMOS (version 21.0, IBM, SPSS, Inc., Chicago, IL, United States) was used to quantify the relationships among soil properties (pH and salt content), soil microbial diversity and soil carbon cycle immobilization-related functional gene diversity. As reported by Burri, the non-significant chi-square value and the root mean square error of approximation were used to assess the goodness of fit (Burri et al., 2018).

## 3 Results

### 3.1 Distribution of carbon cycle immobilization-related microorganisms and functional genes in different salinity gradients

**Figure 1:**
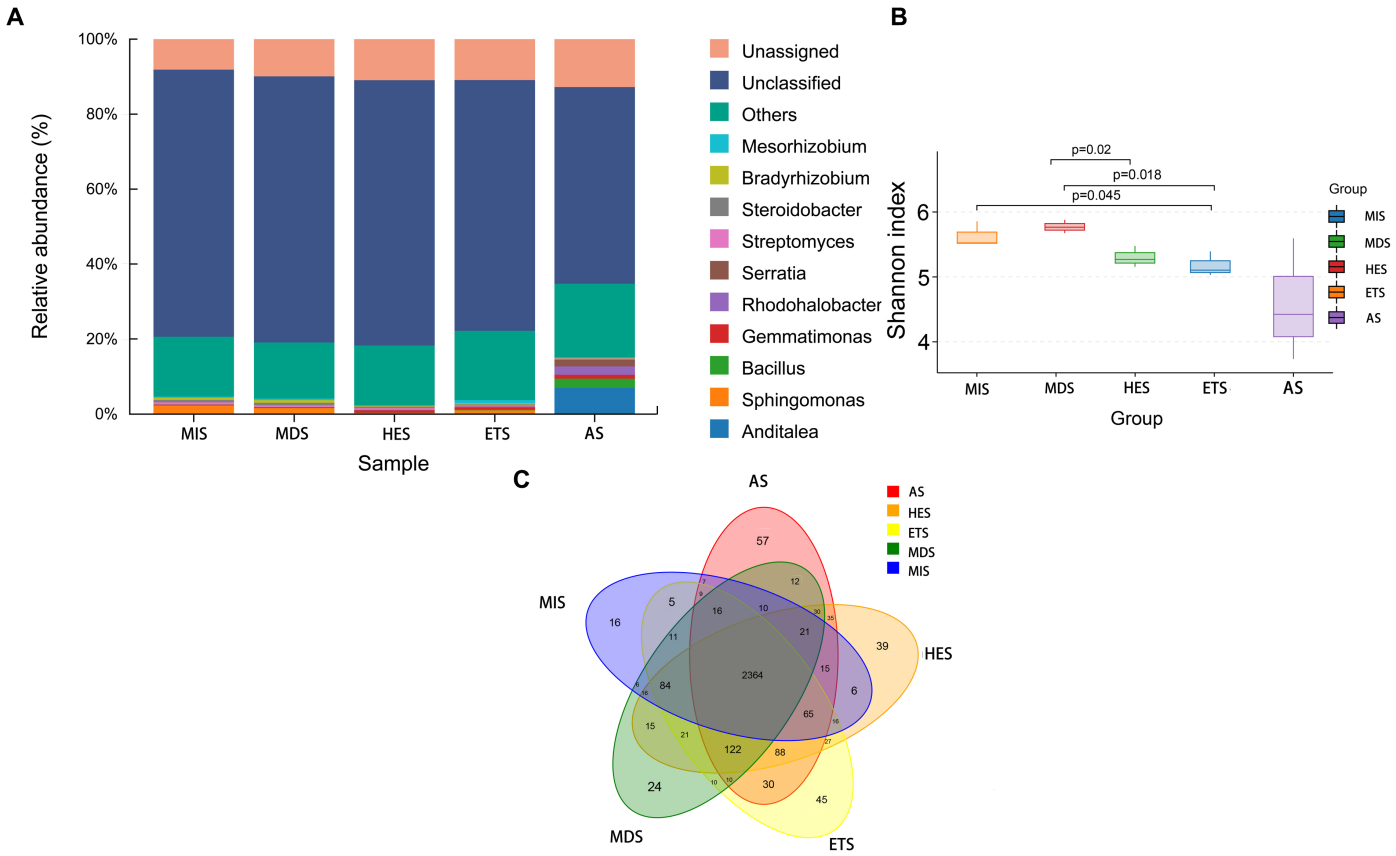
Microbial diversity **(A,B)** and Venn diagram of carbon cycle immobilization-related functional genes in grasslands **(C)** with different salinity gradients. MS, mildly saline grassland; MDS, moderately saline grassland; HES, heavily saline grassland; ETS, extremely saline grassland; AS, alkali spot.

**Figure 2:**
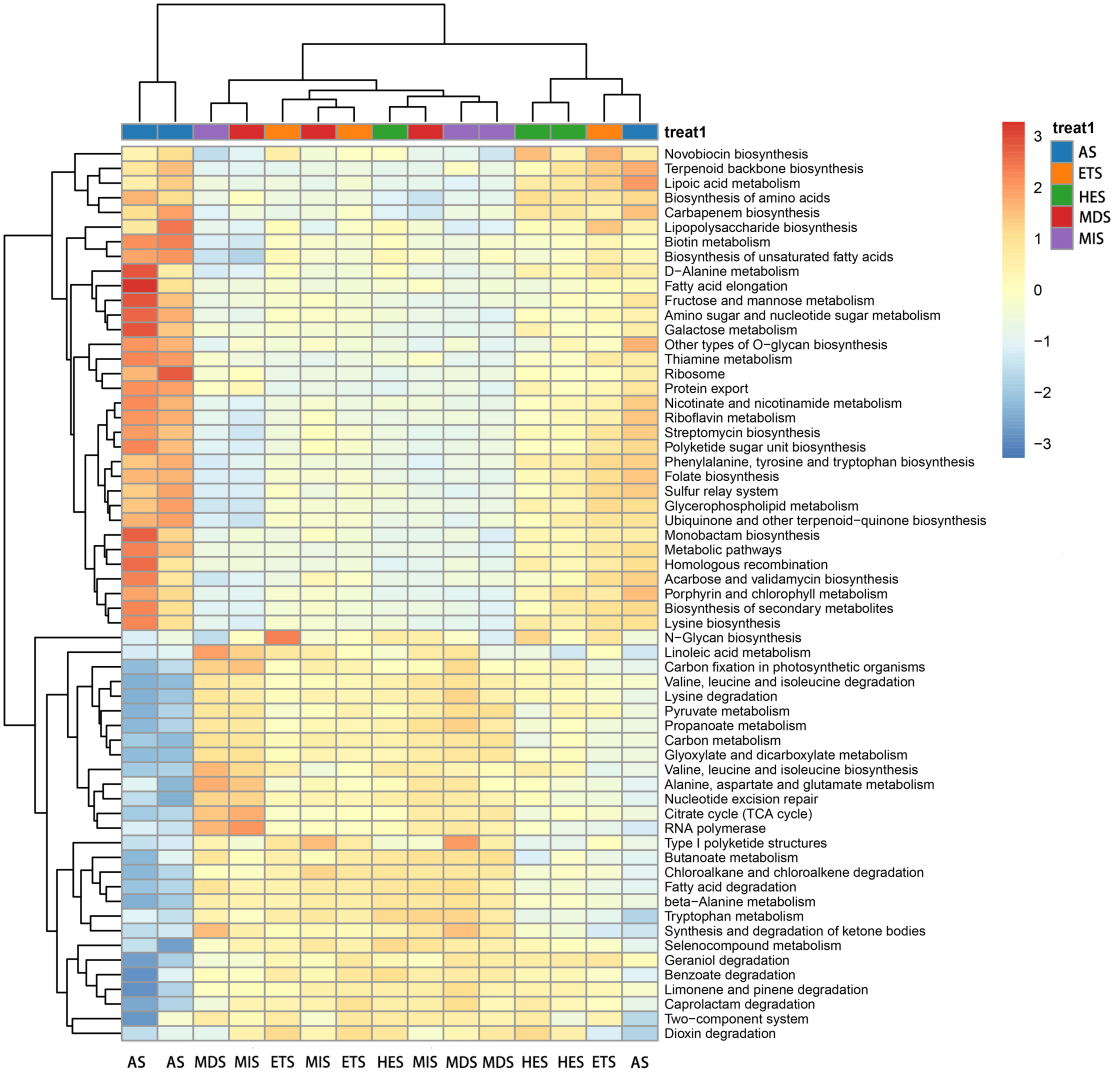
Heat map of differentially expressed functional genes related to the carbon cycle. Positive numbers represent the upregulation of genes, while negative numbers represent the downregulation of genes. MIS, mildly saline grassland; MDS, moderately saline grassland; HES, heavily saline grassland; ETS, extremely saline grassland; AS, alkali spot.

The composition and relative abundance of carbon cycle immobilization-related microorganisms differed in meadow steppe soils with different salinity gradients. The strain composition and abundance were the highest in AS and lowest in HES. In MIS, ETS and MDS, the abundance of *Sphingomonas* species was the highest; it decreased with an increase in salinity and almost disappeared in HES and AS. *Gemmatimonas* species were predominant in HES and exhibited high abundance in ETS and AS. On the other hand, *Anditalea* species were predominant in AS and were less abundant in other salinity gradients. The relative abundance of *Streptomyces* increased with an increase in salinity (Figure 1A). The Shannon index revealed that with an increase in salinity, the diversity of carbon cycle immobilization-related genes decreased. The diversity of carbon cycle immobilization-related genes did not differ significantly between HES and ETS and between MIS and MDS. However, the gene diversity in HES significantly differed from that in MIS and MDS (*p* < 0.05). Similarly, the gene diversity in ETS significantly differed from that in MIS and MDS (*p* < 0.05) (Figure 1B). A Venn map of carbon cycle immobilization-related genes revealed that a total of 2,364 genes exist in five salinity gradients, 57 genes were unique to AS and 16 were unique to MIS. Six genes were detected in MIS and MDS and 27 genes were detected in HES and ETS (Figure 1C). By digital gene expression profiling, 61 differentially expressed functional genes related to the carbon cycle were screened and obtained. The heat map revealed that 33 genes were upregulated in AS, ETS and HES and downregulated in MDS and MIS, with the differences being significant. Of these, genes associated with fatty acid elongation and fructose and mannose metabolism exhibited the most significant differences. Moreover, 28 genes were upregulated in MDS and MIS and downregulated in AS, ETS and HES, with the differences being significant. Of these, genes associated with benzoate, limonene and pinene degradation exhibited the most significant differences (Figure 2).

**Figure 3:**
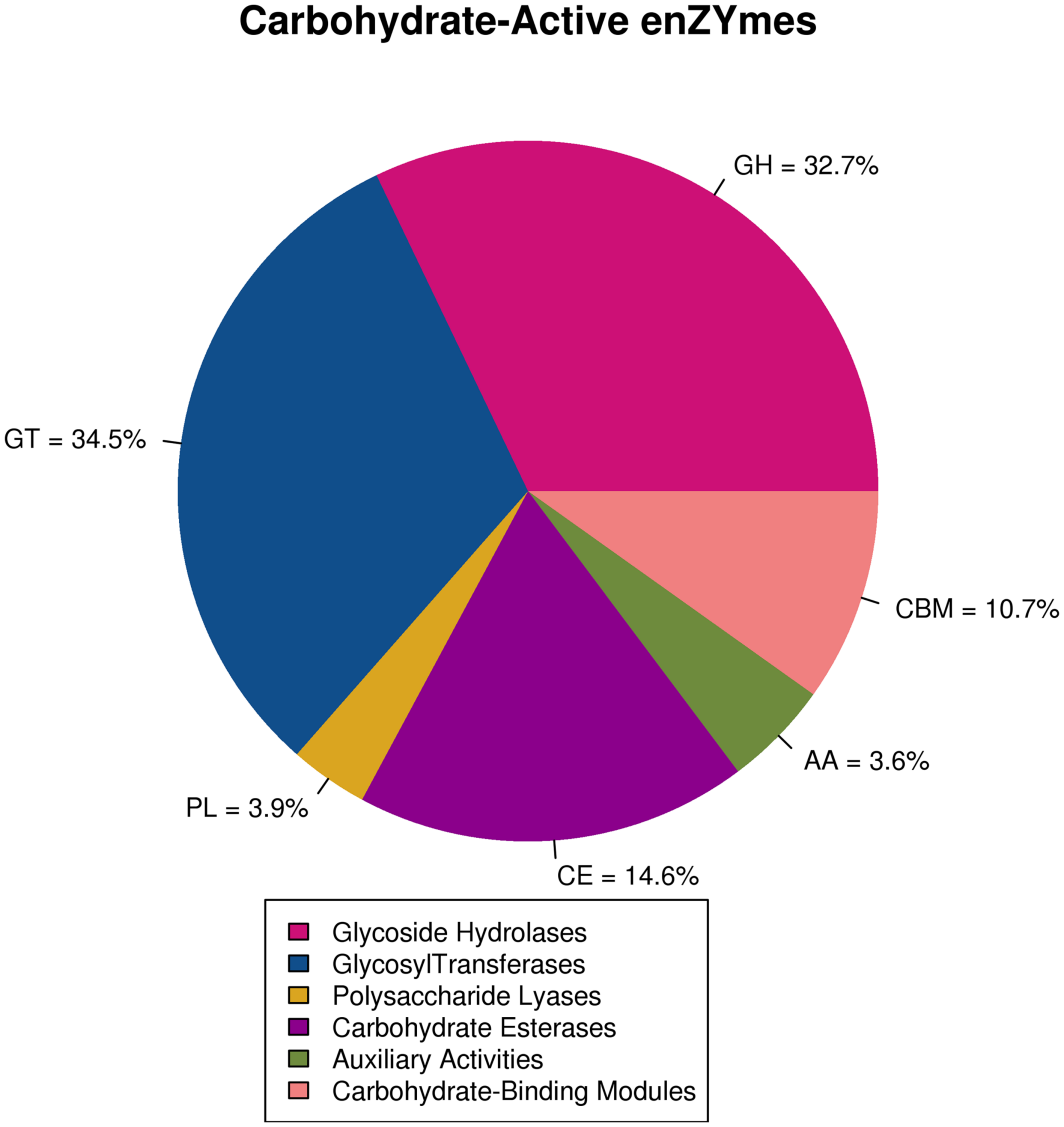
Notes on carbohydrate-active enzymes.

In this study, five CAZymes and 406 different families were significantly annotated (E value <1^e−18^, coverage >0.35) (Supplementary Table S1). The two most representative types of CAZymes were GTs (average 34.5%) and GHs (average 32.7%). CEs were also very abundant (14.6%) (Figure 3). Among GHs, representatives of the GH130 family (often found to have photosporidising activity and an affinity for N-glycans) mainly existed in ETS and HES. The GH3 family (particularly β-glucosidase and β-xylosidase degrading cellulose and hemicellulose, respectively) and the GH55 family (particularly exogenous and endogenous 1,3-glucanases) mainly existed in MDS and MIS (Supplementary Table S1).

**Figure 4:**
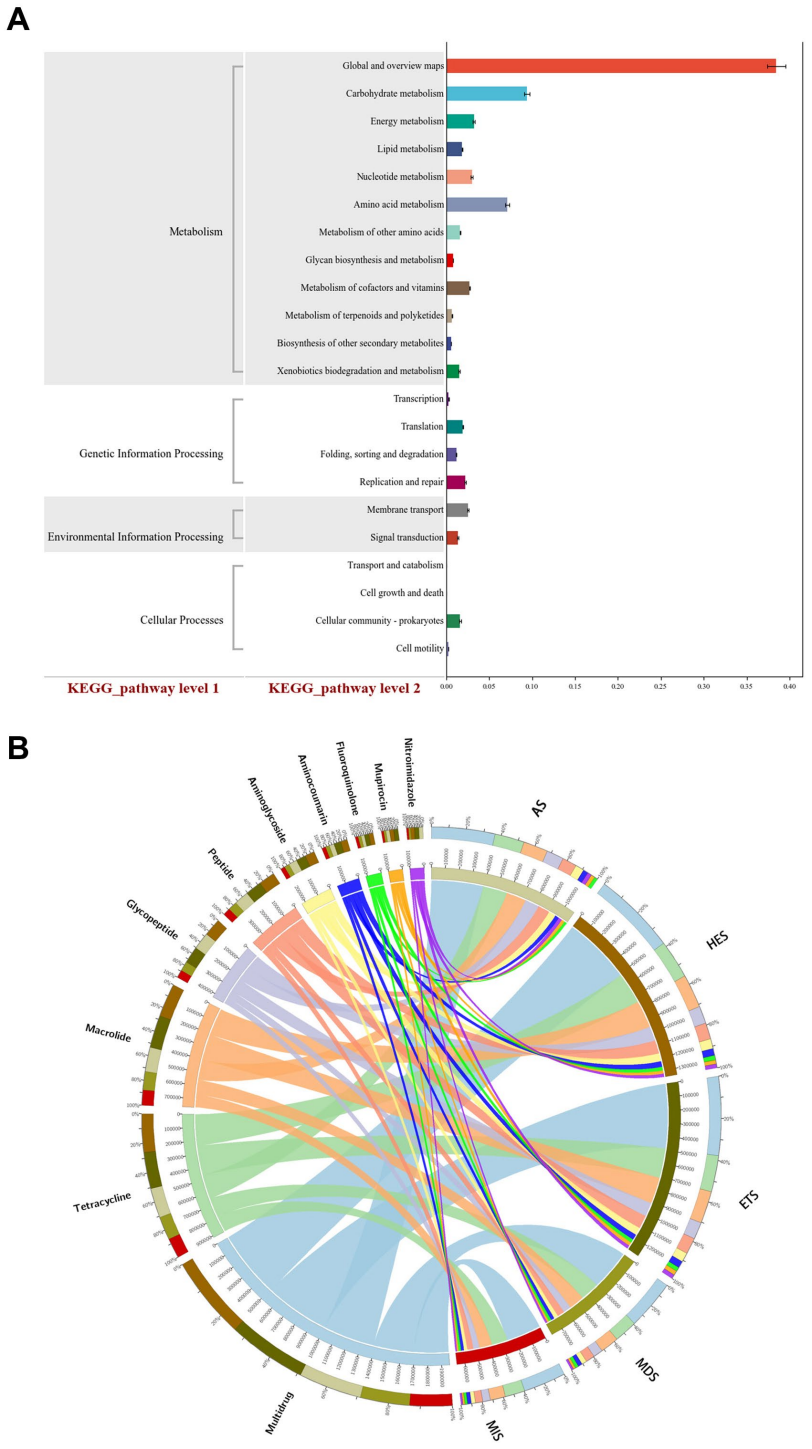
KEGG analysis of carbon cycle immobilization-related functional genes **(A)**. CARD analysis of functional gene composition **(B)**. MIS, mildly saline grassland; MDS, moderately saline grassland; HES, heavily saline grassland; ETS, extremely saline grassland; AS, alkali spot.

To determine the signaling pathway of carbon cycle immobilization-related genes, KEGG analysis of related genes was performed. Among the significantly enriched KEGG pathways, “global and overview maps,” “carbohydrate metabolism,” “amino acid metabolism,” “membrane transport,” “metabolism of vitamins” and “energy metabolism” were signaling pathways of carbon cycle immobilization-related genes. These pathways may play an important role in relevant processes in the carbon cycle (Figure 4A). CARD analysis of carbon cycle immobilization-related genes revealed that genes encoding mupirocins, fluoroquinolones, aminocoumarins, aminoglycosides, peptides, glycopeptides, nitroimidazoles, macrolides, tetracyclines and multidrug were the 10 most significantly enriched carbon cycle immobilization-related genes. Of these, macrolide-encoding genes exhibited a greater correlation with HES and ETS, which had high salinity, while multidrug-encoding genes exhibited a greater correlation with MIS and MDS, which had low salinity. Glycopeptide-and tetracycline-encoding genes exhibited a relatively consistent correlation at each salinity level. With an increase in salinity, the abundance of fluoroquinolone-and aminocoumarin-encoding genes significantly increased (Figure 4B).

### 3.2 Effects of environmental factors on carbon cycle immobilization-related genes in microorganisms

**Figure 5:**
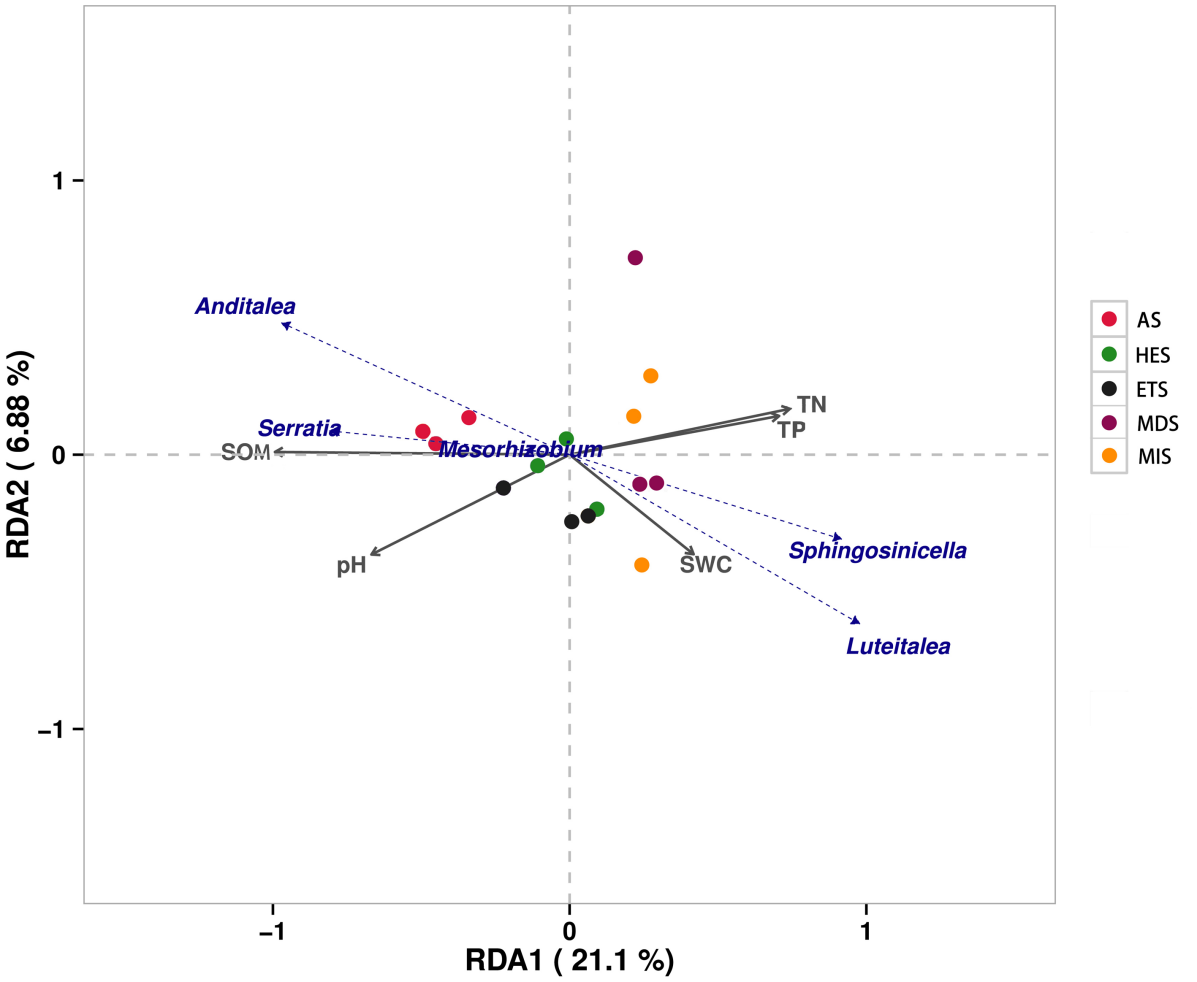
Redundancy analysis of microbial carbon cycle immobilization-related genes in soils with different salinity gradients. MIS, mildly saline grassland; MDS, moderately saline grassland; HES, heavily saline grassland; ETS, extremely saline grassland; AS, alkali spot.

The physicochemical properties of soil significantly varied in saline grasslands having varying gradients of salinity. Soil pH increased with an increase in soil salinity, being the highest in AS at pH 11.56 and lowest in MDS at pH 8.83. SOM content decreased with an increase in salinity. Similarly, SWC tended to decrease with an increase in salinity. Moreover, TN and TP contents were lower in high-salinity grasslands than in low-salinity grasslands (Table 1). Redundancy analysis (RDA) revealed that the composition of carbon cycle immobilization-related genes in AS was very different from that in the other sites, being exclusively distributed in the second quadrant. Moreover, the microorganisms that mainly influenced the composition of carbon cycle immobilization-related functional genes in AS were *Anditalea* and *Serratia* species (Figure 5). The composition of carbon cycle immobilization-related genes was similar in HES and ETS, and *Mesorhizobium* species mainly influenced the composition of carbon cycle immobilization-related functional genes in these gradients. Moreover, MIS and MDS had a similar gene composition, and *Sphingosinicella* and *Luteitalea* species mainly influenced the composition of carbon cycle immobilization-related functional genes in these gradients (Figure 5). In RDA1 and RDA2, SOM and pH had a strong influence on the genetic composition of AS soils, while SWC, TP content and TN content had a strong influence on the genetic composition of HES and ETS soils.

**Table 1:** Analysis of the physicochemical properties of saline grasslands with different salinity gradients.

Sample sites	pH	SOM (g/kg)	SWC (%)	TP (g/kg)	TN (g/kg)	EC (ms/cm)
AS	11.56a	29.84a	26.65a	17.57a	1.03a	3.09a
ETS	10.48b	18.77b	26.19a	11.46b	0.99a	1.69b
HES	10.37b	10.07c	18.99ab	9.12c	0.8a	1.04c
MIS	9.57c	6.38d	17.3b	8.41c	0.17b	0.73c
MDS	8.83d	6.68d	16.08b	6.99d	0.12b	0.28d

Different lowercase letters indicate significant differences between treatments based on the LSD test at *p* ≤ 0.05. Soil organic matter (SOM) content, total nitrogen (TN) content, total phosphorus (TP) content and electrical conductivity (EC).

### 3.3 Carbon cycle immobilization-related genes and the association of microbial composition with soil properties at different salinities

**Figure 6:**
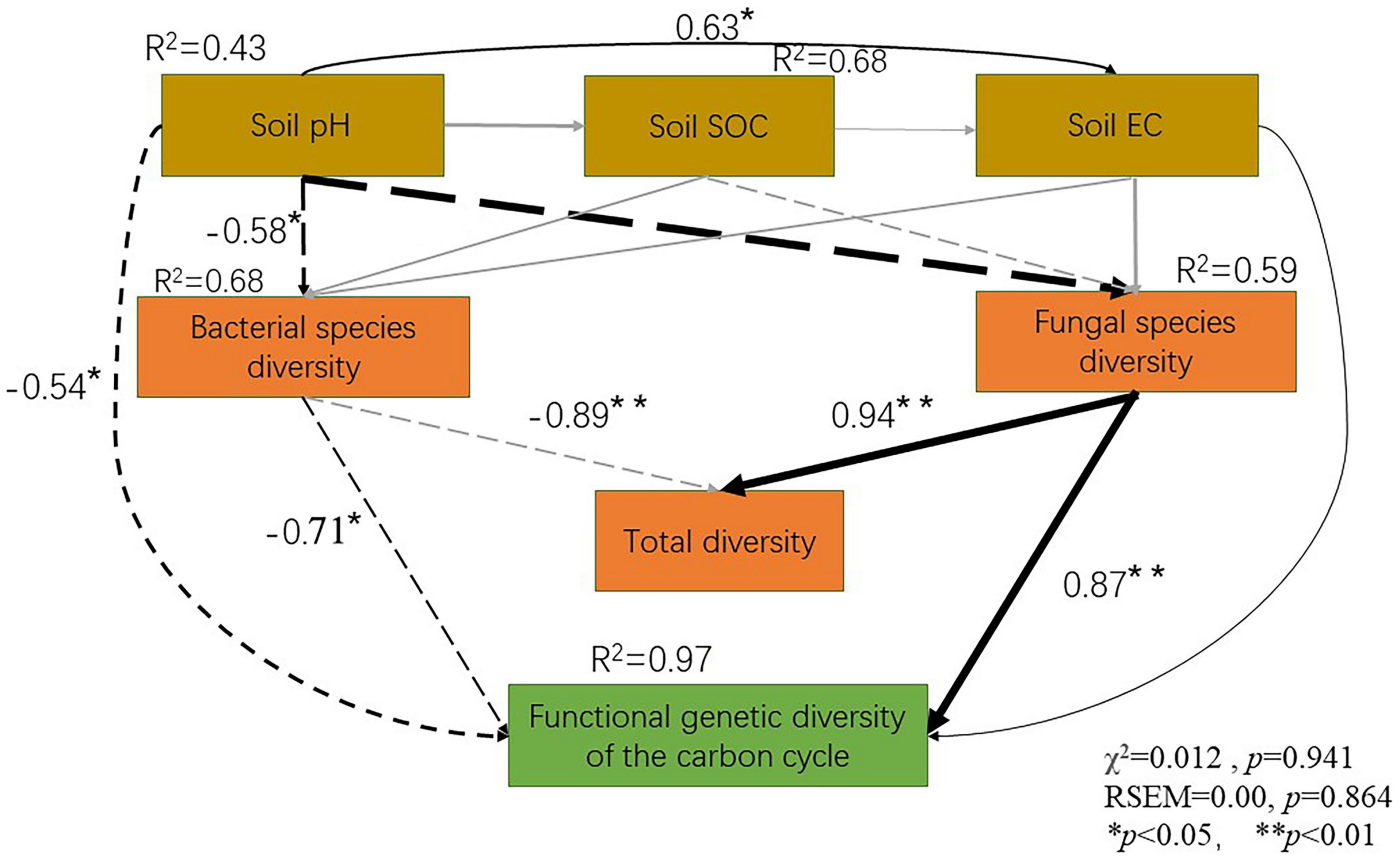
Soil physicochemical properties, microbial species diversity and carbon cycle immobilization-related functional gene composition, as assessed using a structure equation model.

A negative correlation was noted between soil pH and soil bacterial diversity (R^2^ = 0.58, *p* < 0.05). Similarly, soil fungal diversity (R^2^ = 0.89, *p* < 0.01) was negatively correlated with soil pH. However, no significant correlation was noted between soil EC and soil bacterial and fungal diversity. The soil fungal diversity was positively correlated with the total soil diversity (R^2^ = 0.89, *p* < 0.01). However, the soil bacterial diversity (R^2^ = 0.55, *p* < 0.05) was negatively correlated with the total soil diversity (R^2^ = 0.94, *p* < 0.05) and carbon cycle immobilization-related functional gene diversity (R^2^ = 0.97, *p* < 0.05). Moreover, the soil fungal diversity was negatively correlated with the soil functional gene diversity (R^2^ = 0.87, *p* < 0.05). SEM revealed that soil pH and EC had an overall impact on the soil microbial diversity and functional gene diversity. As the soil pH increased, its species diversity and functional gene diversity decreased. However, as the soil EC increased, its total biodiversity and functional gene diversity increased (Figure 6).

## 4 Discussion

### 4.1 Effects of soil pH and EC on carbon immobilization-related genes

Soil salinization leads to a significant increase in soil pH and EC (Lu et al., 2022). In general, elevated soil pH enhances the metabolic and decomposition activities of microorganisms, leading to increased soil carbon loss, lower soil organic carbon content, and reduced genetic diversity associated with carbon cycle fixation (Tang et al., 2019). Therefore, the organic carbon content and gene diversity associated with carbon cycle fixation in severely salinized soils were generally lower than those in mild salinized soils (Li L. F. et al., 2021; Li C. Y. et al., 2021; Li B. et al., 2021). The negative effects of salinization on soil microbial and carbon cycling-related genes can be explained by the low osmotic potential caused by high concentrations of salt, which can reduce the water supply for microbial activity, lead to soil microbial decomposition, and reduce soil microbial diversity (Zhou et al., 2021). The abundance of microbial carbon fixation genes indicates the carbon sequestration potential of microorganisms, and the higher the gene abundance, the stronger the corresponding carbon sequestration potential, and the greater the contribution to soil carbon sequestration. Decreased genetic diversity associated with carbon cycling sequestration indicates a decrease in the carbon sequestration potential of soil microorganisms (Xu et al., 2021).

In our study, soil pH and EC also increased significantly as the salinity gradient increased, and the increase in soil pH reduced the microbial abundance associated with soil carbon cycling and the diversity of functional genes associated with carbon cycling fixation. Redundancy analysis showed that soil Anditalea and serratia were significantly positively correlated with soil SOM and negatively correlated with soil pH, indicating that the abundance of microbial diversity and carbon fixation functional genes were closely related to soil pH and organic matter (Figure 5). Therefore, the microbial abundance of EST soil was significantly lower than that of MIS and MDS soils, which may be due to the lower SOM content and higher pH. At the same time, the low abundance of EST carbon-fixing functional genes may also be related to the inhibition of pH and EC in soil, and these results are consistent with previous studies. In addition, it has also been suggested that the growth of some carbon-sequestration bacteria in high-saline soils is inhibited due to higher oxygen concentrations, resulting in a weak microbial carbon sequestration potential in high-saline soils (Balasubramanian et al., 2020).

### 4.2 Effects of grassland salinity on carbon cycle immobilization-related genes

The main determinants of the rate of carbon cycle immobilization-related processes are the availability and accessibility of substrates and the activity of microorganisms (Ma et al., 2021). Soil salinization directly affects the decomposition process of soil microorganisms, microbial activities and the growth and turnover of microbial communities. Severe salinization leads to gradual substrate depletion (Li L. F. et al., 2021; Li C. Y. et al., 2021; Li B. et al., 2021). Substrate limitation, low microbial biomass and decreased high-quality specific microbial activity can reduce microbial carbon immobilization, resulting in the loss of carbon from the system; this has a potential positive feedback effect on soil salinization (Li et al., 2019).

We observed that the diversity of genes and enzyme families associated with the carbon cycle decreased during soil salinization. However, no significant difference was noted in the diversity between MDS and MIS with low salinity and between ETS and HET with high salinity. However, the diversity in ETS and HET was significantly different from that in MDS and MIS. This observation is consistent with the change in SOM and microbial biomass per unit, which is also associated with a change in grassland salinity (Jiang et al., 2021). Although the measurement of potential enzyme activity is a general indicator, it reflects the activity of a limited group of enzymes under optimal conditions, thereby providing an incomplete understanding of what happens under natural conditions (Chen et al., 2020).

Among CAZymes, the abundance of GHs and GTs was higher and decreased with an increase in salinity. This indicated that increased salinity negatively affected the grassland soil carbon cycle, which was consistent the change trend noted in soil organic carbon contents (Song et al., 2019). Moreover, physiological changes, especially the ability to produce and secrete the types of enzymes widely existing in soil microorganisms, were found to be an important reason for grassland soil salinization (Wang et al., 2020). The increase in *Gemmatimonas* and decrease in *Sphingomonas* and *Streptomyces* with an increase in salinity exhibited the genes involved in the degradation of all organic carbon forms, indicating a group-specific response of grassland soil microorganisms to grassland salinization and/or substrate preference (Lv et al., 2020; Liang et al., 2021).

In this study, we only sampled during the annual grassland growth period, and there was a lack of multi-season samples, which had certain limitations, and some articles showed that there were different differences in microbial diversity and carbon fixation-related functional genes in saline-alkali grassland under different seasons (Taylor et al., 2021), and then we will study the direction of seasonal changes in microbial diversity and carbon fixation-related functional genes in Songnen saline-alkali grassland, which will have a significant impact on the protection and restoration of Songnen saline-alkali grassland. Soil carbon management and other aspects provide a deeper theoretical basis.

## 5 Conclusion

In this study, soil metagenomics revealed the mechanisms of microbial diversity and functional gene diversity, which are the main processes of carbon cycling under soil salinization in different grasslands. We found that the microbial community composition and carbon cycle immobilization-related functional gene composition in saline–alkaline in the Songnen Plain in Northeast China were closely and negatively related to soil pH and salinity. Finally, we found that soil salinization lowered the expression levels of genes and enzyme families involved in the immobilization of carbon-rich polymers. Therefore, we believe that improving the functional gene diversity of microorganisms and carbohydrate-related enzyme activities are the key factors to support the improvement and restoration of soil carbon cycle in saline-alkali grassland.

## Data availability statement

The data presented in the study are deposited in the NCBI repository under accession number PRJNA1074398.

## Author contributions

HX: Investigation, Writing – original draft. YW: Data curation, Writing – original draft. XSu: Writing – original draft. XSo: Writing – original draft. JL: Writing – original draft. ZB: Writing – original draft. GH: Writing – original draft. LQ: Funding acquisition, Project administration, Resources, Writing – original draft, Writing – review & editing.

## Footnotes

^1^ http://www.usadellab.org/cms/index.php?page=trimmomatic
